# National Surveillance Study on Carbapenem Non-Susceptible *Klebsiella pneumoniae* in Taiwan: The Emergence and Rapid Dissemination of KPC-2 Carbapenemase

**DOI:** 10.1371/journal.pone.0069428

**Published:** 2013-07-24

**Authors:** Sheng-Kang Chiu, Tsu-Lan Wu, Yin-Ching Chuang, Jung-Chung Lin, Chang-Phone Fung, Po-Liang Lu, Jann-Tay Wang, Lih-Shinn Wang, L. Kristopher Siu, Kuo-Ming Yeh

**Affiliations:** 1 Division of Infectious Diseases and Tropical Medicine, Department of Medicine, Tri-Service General Hospital, National Defense Medical Center, Taipei, Taiwan, ROC; 2 Graduate Institute of Medical Sciences, National Defense Medical Center, Taipei, Taiwan, ROC; 3 Department of Laboratory Medicine, Chang Gung Memorial Hospital, Kweishan, Taoyuan, Taiwan, ROC; 4 Department of Medical Research, Chi Mei Medical Center, Tainan Hsien, Taiwan, ROC; 5 Section of Infectious Diseases, Department of Medicine, Taipei Veterans General Hospital, National Yan-Ming University, Taipei, Taiwan, ROC; 6 Department of Internal Medicine, Kaohsiung Medical University Hospital, Kaohsiung, Taiwan, ROC; 7 Division of Infectious Diseases, Department of Medicine, National Taiwan University Hospital, Taipei, Taiwan, ROC; 8 Department of Infectious Diseases, Buddhist Tzu Chi General Hospital, Hualien, Taiwan, ROC; 9 Institute of Infectious Diseases and Vaccinology, National Health Research Institutes, Miaoli, Taiwan, ROC; National Institutes of Health, United States of America

## Abstract

**Objectives:**

The global spread and increasing incidence of carbapenem non-susceptible *Klebsiella pneumoniae* (CnSKP) has made its treatment difficult, increasing the mortality. To establish nationwide data on CnSKP spread and carbapenem-resistance mechanisms, we conducted a national surveillance study in Taiwanese hospitals.

**Methods:**

We collected 100 and 247 CnSKP isolates in 2010 and 2012, respectively. The tests performed included antibiotic susceptibility tests; detection of carbapenemase, extended-spectrum β-lactamases (ESBL), and AmpC β-lactamases genes; outer membrane porin profiles; and genetic relationship with pulsed-field gel electrophoresis and multilocus sequence type.

**Results:**

The resistance rate of CnSKP isolates to cefazolin, cefotaxime, cefoxitin, ceftazidime, and ciprofloxacin was over 90%. Susceptibility rate to tigecycline and colistin in 2010 was 91.0% and 83.0%, respectively; in 2012, it was 91.9% and 87.9%, respectively. In 2010, carbapenemase genes were detected in only 6.0% of isolates (4 *bla*_IMP-8_ and 2 *bla*_VIM-1_). In 2012, carbapenemase genes were detected in 22.3% of isolates (41 *bla*_KPC-2_, 7 *bla*_VIM-1,_ 6 *bla*_IMP-8,_ and 1 *bla*_NDM-1_). More than 95% of isolates exhibited either OmpK35 or OmpK36 porin loss or both. Impermeability due to porin mutation coupled with AmpC β-lactamases or ESBLs were major carbapenem-resistance mechanisms. Among 41 KPC-2-producing *K. pneumoniae* isolates, all were ST11 with 1 major pulsotype.

**Conclusions:**

In 2010 and 2012, the major mechanisms of CnSKP in Taiwan were the concomitance of AmpC with OmpK35/36 loss. KPC-2-KP dissemination with the same ST11 were observed in 2012. The emergence and rapid spread of KPC-2-KP is becoming an endemic problem in Taiwan. The identification of NDM-1 *K. pneumoniae* case is alarming.

## Introduction

*Klebsiella pneumoniae* is an important pathogen causing various kinds of infection including bacteraemia, pneumonia, liver abscess, and urinary tract infections [1], [2]. The spread of carbapenem non-susceptible *Klebsiella pneumoniae* (CnSKP) has made its treatment difficult and caused higher disease-related mortality [3], [4]. The incidence of carbapenem resistance among *K. pneumoniae* in intensive care units in Taiwan increased from 1.2% in 2003 to 11.9% in 2011 [5]. Carbapenem resistance in *K. pneumoniae* can arise due to the presence of true carbapenemase, or via the combination of impermeability and production of extended-spectrum beta-lactamase (ESBL) or a strongly expressed AmpC enzyme [6], [7]. However, the mechanisms involved in the presence of CnSKP isolates in Taiwan have not been fully investigated.

The carbapenemase enzyme found in *K. pneumoniae* could be clavulanic-acid-inhibited β-lactamases (Ambler class A families: KPC, NMC, IMI, SME, and GES), metallo-β-lactamases (Ambler class B families: IMP, VIM, NDM-1, GIM, SPM, and SIM), or expanded-spectrum oxacillinases (Ambler class D family: OXA-48) [8]. Carbapenemase-producing *K. pneumoniae* (KPC-KP) strains were first reported in 2001 from strain isolated in 1996 in the USA [9]. Since then, they have been identified in many places around the world [8], [10]–[13]. KPC-KP was not reported in Taiwan until 2011 [14], and it could be an emerging threat to clinical practice in Taiwan. Thus, continuous surveillance of antimicrobial resistance is warranted.

CnSKP that exhibits multidrug resistance reduces therapeutic choices and may lead to untreatable infections [8]. Therefore, identification of carbapenemase genes using molecular techniques may help us understand the spread of CnSKP and assist in determining appropriate control measures. In this study, we conducted a nationwide, multicentre prospective surveillance study in Taiwanese hospitals in 2010 and 2012.

## Materials and Methods

### Hospital Settings

In 2010, 8 medical centres from around Taiwan were enrolled in the study. These included the Keelung Chang Gung Memorial Hospital, Tri-Service General Hospital, Taipei Veterans General Hospital, and Linkou Chang Gung Memorial Hospital in the north; the China Medical University Hospital and Chiayi Chang Gung Memorial Hospital in the central region of the country; and the Kaohsiung Chang Gung Memorial Hospital and Kaohsiung Medical University Hospital in the south. In 2012, the number of participating hospitals was extended to 17, including 9 medical centres and 8 regional hospitals. The hospitals added in 2012 included the National Taiwan University Hospital, Taoyuan Armed Forces General Hospital, Buddhist Tzu Chi General Hospital, Hualien Armed Forces General Hospital, National Yang-Ming University Hospital, Taichung Armed Forces General Hospital, Chi Mei Medical Center, Kaohsiung Armed Forces General Hospital, and Kaohsiung Municipal Hsiaokang Hospital.

### Bacterial Isolates

From January through December 2010 and from January through December 2012, CnSKP isolates were collected from clinical specimens sent for culture in the microbiological laboratories of participating hospitals. A single strain was selected per patient. Carbapenem non-susceptibility was defined as a minimum inhibitory concentration (MIC) of at least 2 mg/L for imipenem or meropenem. The isolates collected from each hospital were sent to the National Health Research Institutes, Miaoli, Taiwan and were stored at −70°C in 10% glycerol Luria-Bertani medium before analysis. Species confirmation was performed by standard biochemical methods, on a VITEK 2 automated system (bioMérieux, Marcy l’Etoile, France).

### Antimicrobial Susceptibility Testing

MICs for carbapenems (ertapenem, imipenem, meropenem, and doripenem) and other antimicrobial agents [cefazolin, cefotaxime, cefoxitin, cefepime, ciprofloxacin, amikacin, gentamicin, trimethoprim-sulfonamides (SXT), and colistin] were determined by broth microdilution method (Sensititre, Trek Diagnostic Systems, Cleveland, OH, USA). The Clinical and Laboratory Standards Institute (CLSI) M100-S22 interpretive breakpoints were used to interpret the MIC results for all antimicrobial agents studied, except tigecycline and colistin [15]. The MICs for tigecycline were determined using the E-test (AB Biodisk, Solna, Sweden) on Mueller-Hinton media, and susceptibility to tigecycline was defined based on the Food and Drug Administration criteria (MIC ≤2 mg/L) [16]. The susceptibility to colistin was defined based on the European Committee on Antimicrobial Susceptibility Testing criteria (MIC ≤2 mg/L) [17].

### Detection of Genes Encoding Carbapenemase, AmpC β-lactamase, and ESBLs

CnSKP isolates were subjected to polymerase chain reaction (PCR) detection of carbapenemase genes (encoding Ambler class A families KPC, NMC, IMI, SME, and GES; Ambler class B families IMP, VIM, NDM, GIM, SPM, and SIM; and Ambler class D family OXA-48-type) [18], plasmid-borne AmpC-like genes (encoding CMY, and DHA) [19], and ESBL genes (encoding CTX-M [20], TEM [20], and SHV [21]). The primers are listed in Table S1. Bacterial DNA was prepared by suspending 1 loop of freshly cultured cells in 500 µL of sterile distilled water and heating the mixture at 95°C for 10 min. The amplification conditions were as follows: 95°C for 5 min, followed by 35 cycles of 95°C for 1 min; 54°C for 1 min; and 72°C for 1 min, with a single, final, elongation step at 72°C for 10 min.

The amplicons were sequenced and the entire sequences were compared were compared to the National Center for Biotechnology Information (NCBI) database at www.ncbi.nlm.gov/blast/to determine the molecular type. Nucleotide sequencing was conducted using corresponding primers specific to *bla*_SHV_, *bla*_CTX-M_, *bla*_TEM_, *bla*_CMY_, *bla*_DHA_, *bla*_KPC_ and *bla*_NDM_ genes with an ABI Prism 3700 DNA sequencer (Applied Biosystems, Foster City, CA, USA).

### Identification of Outer Membrane Porins (OmpK35 and OmpK36)

Isolates were grown in high-osmolarity Mueller-Hinton broth to the logarithmic phase and were lysed by sonification. Outer membrane porins (OMPs) were isolated according to the rapid procedure of Carlone *et al* [22]. The OMP profiles were identified by 12% sodium dodecyl sulphate-polyacrylamide gel electrophoresis (SDS-PAGE) followed by coomassie blue staining (Gibco-BRL, Grand Island, NY, USA). *K. pneumoniae* ATCC 13883 was used as the control strain.

### Pulsed-Field Gel Electrophoresis

Pulsed-field gel electrophoresis (PFGE) was performed for the KPC-KP isolates. In brief, bacterial chromosomal DNAs were digested by using *Xba*I (New England Biolabs, Beverly, MA, USA) [23]. Electrophoresis was carried out for 22 h at 14°C with pulse times ranging from 2 to 40 s at 6 V/cm with Bio-Rad CHEF MAPPER apparatus (Bio-Rad Laboratories, Richmond, CA, USA). A dendrogram based on the unweighted pair group was generated by the methods previously described [24]. Isolates that had more than 80% similarity on the PFGE profiles were considered as closely related strains.

### Multilocus Sequence Type

Multilocus sequence type (MLST) was performed on all isolates from 2010 and KPC-KP in 2012 according to the protocol described on the *K. pneumoniae* MLST website (http://www.pasteur.fr/recherche/genopole/PF8/mlst/Kpneumonia.html). MLST results were typed according to the international *K. pneumoniae* MLST database created in 2005 at the Pasteur Institute in Paris, France [25].

### Statistical Analysis

Statistical analyses were performed using SPSS software package (version 16, Chicago, IL, USA). Analysis was performed by the chi-square test or Fisher’s exact test for categorical variables. A value of p<0.05 was considered statistically significant.

## Results

### Characteristics of Bacterial Isolates

A total of 100 single-patient isolates were collected in 2010, of which 67 were from northern, 16 were from central, 17 were from southern Taiwan. A total of 247 single-patient isolates were collected in 2012, of which 206 were from northern, 19 were from central, 20 were from southern, and 2 were from eastern Taiwan (Table 1).

**Table 1:** Regional distributions of the participating hospitals, carbapenem non-susceptible *K. pneumoniae* isolates, and *K. pneumoniae* carbapenemase-2-producing *K. pneumoniae.*

Location	Number of participating hospitals		CRKP isolates		Serving population^a^	No. of KPC-2 isolates
	2010	2012	2010	2012		
**North**	4	6	67	206	∼8,265,000	38
**West**	2	3	16	19	∼5,766,000	1
**South**	2	5	17	20	∼6,325,000	2
**East**	0	3	0	2	∼1,024,000	0
**Total**	8	17	100	247	∼21,380,000	41

a Population was presented according to the data of National Statics, ROC (Taiwan) in 2012.

### Antimicrobial Susceptibility of CnSKP Isolates

The MIC ranges, MIC_50_ values, and MIC_90_ values of 15 antimicrobial agents as well as the rates of susceptibility to said agents against the CnSKP isolates are listed in Table 2. Among the isolates collected in 2010, all isolates (100/100) were resistant to cefazolin, cefoxitin, and ertapenem. Susceptibility rates to cefotaxime and ceftazidime were 1.0%. The susceptibility rates to cefepime, imipenem, and meropenem were less than 10%. Susceptibility rates to gentamicin and amikacin were 11.0% (11/100) and 22.0% (22/100), respectively. The susceptibility rate to ciprofloxacin was 7%. The susceptibility rate to SXT was 10.0% (10/100). Susceptibility rates to tigecycline and colistin were 91.0% (91/100) and 83.0% (83/100), respectively.

**Table 2:** Results of the antimicrobial susceptibility tests for carbapenem non-susceptible *K. pneumoniae* isolates collected in 2010 and 2012.

Antibiotics	2010 (N = 100 isolates)						2012 (N = 247 isolates)				p value
	MIC (mg/L)			Susceptibility^ a^			MIC (mg/L)			Susceptibility^a^	
	Range	50	90		n (%)	Range		50	90	n (%)	
Ertapenem	1−≥8	≥8	≥8		0 (0.0)	≤0.25−≥8		≥8	≥8	2 (0.8)	1.000
Imipenem	0.5−≥8	≥8	≥8		4 (4.0)	0.5−≥8		≥8	≥8	3 (1.2)	0.109
Meropenem	≤0.25−≥8	≥8	≥8		8 (8.0)	≤0.25−≥8		≥8	≥8	66 (26.7)	<0.001
Doripenem	0.25−≥4	≥4	≥4		15 (15.0)	≤0.12−≥4		≥4	≥4	63 (25.5)	0.034
Amikacin	≤4−≥32	≥32	≥32		22 (22.0)	≤4−≥32		≤4	≥32	163 (66.0)	<0.001
Gentamicin	≤1−≥16	≥16	≥16		11 (11.0)	≤1−≥16		≥16	≥16	109 (44.1)	<0.001
Cefazolin	≥32	≥32	≥32		0 (0.0)	≥32		≥32	≥32	0(0.0)	NA
Cefotaxime	≤1−≥64	≥64	≥64		1 (1.0)	≥64		≥64	≥64	0 (0.0)	0.288
Cefoxitin	≥32	≥32	≥32		0 (0.0)	≥32		≥32	≥32	0 (0.0)	NA
Ceftazidime	4−≥32	≥32	≥32		1 (1.0)	≥32		≥32	≥32	0 (0.0)	0.288
Cefepime	≤1−≥32	≥32	≥32		6 (6.0)	≤1−≥32		≥32	≥32	27 (10.9)	0.156
Ciprofloxacin	0.25−≥4	≥4	≥4		7 (7.0)	≤0.06−≥4		≥4	≥4	21 (8.5)	0.642
Tigecycline	≤0.25−16	1	2		91 (91.0)	≤0.25−16		0.5	2	227 (91.9)	0.783
Colistin	1−≥4	2	≥4		83 (83.0)	≤0.5−≥4		≤0.5	≥4	217 (87.9)	0.231
SXT^b^	≤0.5−≥16	≥16	≥16		10 (10.0)	≤0.5−≥16		≥16	≥16	46 (18.6)	0.048

^a^Susceptibility was interpreted by using the CLSI 2012 criteria.

b Trimethoprim/sulfamethoxazole and MIC was presented according to the concentration of trimethoprim.

Most of the antimicrobial resistance patterns in isolates collected in 2012 were similar to those observed in isolates collected in 2010. Exceptions to this were the susceptibility rates to gentamicin (44.1% vs. 11.0%, p<0.001), amikacin (66.0% vs. 22.0%, p<0.001), meropenem (26.7% vs. 8.0%, p<0.001), doripenem (25.5% vs. 15.0%, p  = 0.034), and SXT (18.6% vs. 10.0%, p  = 0.048), which were higher than those observed in 2010. The susceptibility rates to tigecycline and colistin in 2012 were 91.9% (227/247) and 87.9% (217/247), respectively.

Susceptibility rates to amikacin (62.6% vs 22.0%, p<0.001), gentamicin (43.9% vs 11.0%, p<0.001), and SXT (20.0% vs 10.0%, p<0.034) were higher in isolates collected in 2012 from original 8 hospitals than isolates collected in 2010. Susceptibility rates to meropenem (11.0% vs 53.5%, p<0.001) and doripenem (8.4% vs 54.3, p<0.001) were lower in isolates collected in 2012 from 8 original hospitals than isolates collected in 2012 from 9 newly participating hospitals.

### Detection of Genes Encoding Carbapenemase, AmpC β-lactamase, and ESBLs

Carbapenemase genes were detected in only 6.0% (6/100) of isolates collected in 2010. Four of these isolates expressed *bla*_IMP-8_; the other 2 isolates expressed *bla*_VIM-1_. All other isolates were negative for all carbapenemases tested. Seventy-eight isolates exhibited Amp-C β-lactamase: 76 isolates had *bla*_DHA-1_ and 2 isolates had *bla*_CMY-2_. Among the isolates with genes encoding ESBLs, 42 isolates exhibited the CTX-M-9 group (*bla*_CTX-M-14_), 4 isolates exhibited the CTX-M-1 group (2 isolates exhibited *bla*_CTX-M-3_, 2 isolates exhibited *bla*_CTX-M-15_). Twenty-one isolates harboured the SHV-type ESBLs genes (*bla*_SHV-2_, *bla*_SHV-2A_, *bla*_SHV-5_, *bla*_SHV-12_, *bla*_SHV-28_, and *bla*_SHV-31_).

Carbapenemase genes were detected in 22.3% of isolates collected in 2012 (55/247). Further, *bla*_NDM-1_ was detected in 1_,_ *bla*_IMP-8_ in 6, *bla*_VIM-1_ in 7, and *bla*_KPC-2_ in 41 isolates. No isolates harboured the *bla*_IMI_, *bla*_SME_, *bla*_GES_, *bla*_NMC_ and *bla*_OXA-48-type_ genes. However, 70.8% (175/247) of isolates exhibited Amp-C β-lactamase: 155 and 15 isolates harboured the *bla*_DHA-1_ and *bla*_CMY-2_ genes, respectively, and 5 isolates carried both *bla*_DHA-1_ and *bla*_CMY-2_. Among the isolates with genes encoding ESBLs, 157 exhibited the CTX-M-9 group (*bla*_CTX-M-14_), 12 exhibited the CTX-M-1 group, and 4 carried both the CTX-M-9 and CTX-M-1 groups. Forty-seven isolates harboured SHV-type ESBLs genes (*bla*_SHV-2_, *bla*_SHV-2A_, *bla*_SHV-5_, *bla*_SHV-12_, and *bla*_SHV-120_).

Thirty-eight of the 41 KPC-2-KP isolates were from northern Taiwan, 2 were from southern Taiwan, and 1 was from central Taiwan.

### Analysis of the OMP Profiles

SDS-PAGE analysis of the isolates from 2010 revealed that 71.0% (71/100) of isolates lacked both OmpK35 and OmpK36 porins, followed by 18.0% (18/100) of isolates without the OmpK35 porin and 9.0% (9/100) of isolates without the OmpK36 porin. Among the isolates collected in 2012, 36.4% (90/247) lacked both the OmpK35 and OmpK36 porins, followed by 55.9% (138/247) of isolates that lacked the OmpK35 porin and 2.4% (6/247) of isolates without the OmpK36 porin. Further, 5.3% (13/247) of isolates did not show outer membrane porin loss. In all, 95.7% of isolates (332/347) exhibited defects either in OmpK35 or in OmpK36. Combination of outer membrane porin profiles with carbapenemase, AmpC β-lactamase, or ESBLs is shown in Table S2.

### PFGE Analysis and MLST

PFGE was performed on 100 isolates collected in 2010, and the dendrogram (Figure S1) revealed that there was no dominant clone and inter-hospital spread in 2010. Several intra-hospital clonal spreads were detected by using the 80% similarity as the cut-off.

**Figure 1:**
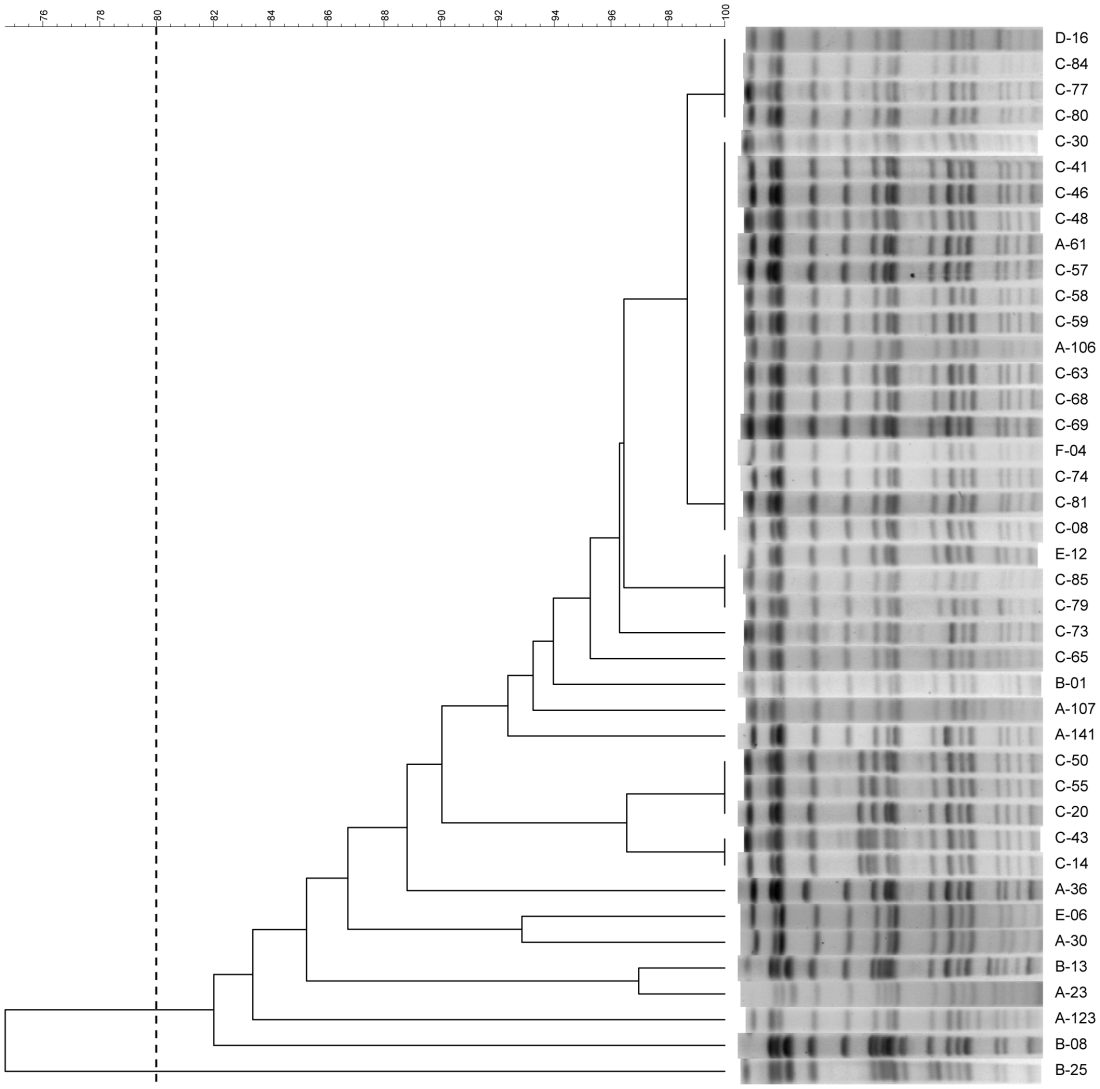
Pulsed-field gel electrophoresis profiles and dendrogram. Pulsed-field gel electrophoresis profiles and dendrogram of the 41 KPC-2-producing *K. pneumoniae* isolates. One major pulsotype is shown using the 80% similarity as the cut-off. All these KPC-2-producing *K. pneumoniae* isolates are ST11.

A dendrogram of the 247 isolates collected in 2012 is shown in Figure S2. The dendrogram and band patterns of KPC-2-producing *K. pneumoniae* strains are shown in Figure 1. One major pulsotype were identified using the 80% similarity as the cut-off on PFGE profiles. There was a clonal spread of 16 identical isolates obtained from 3 hospitals.

MLST typing was performed on the 100 isolates collected in 2010 and on the 41 KPC-2-KP isolates in 2012. Among the 2010 isolates, 59.0% (59/100) were sequence type (ST) 11, 6.0% were ST15 (6/100), 7.0% were ST37 (7/100), 5% were ST1 (5/100), 4% were ST524 (4/100), 3% were ST48 (3/100), and 2% were ST147 (2/100). Other ST types had only 1 isolate each. All 41 isolates with the KPC-2 gene in 2012 were ST11.

## Discussion

Until now, no national data specifically on CnSKP in Taiwan has been collected and studied. The present report showed that the major mechanism underlying the CnSKP phenotype in Taiwan was the loss of outer membrane porins combined with β-lactamases such as AmpC enzyme (*bla*_DHA-1_ and *bla*_CMY-2_) or ESBLs (*bla*_SHV-12_, *bla*_CTX-M_). A low burden of carbapenemase production among CnSKP was found in Taiwan in 2010, with only 6.0% (6/100) of CnSKP isolates being metallo-β-lactamase (*bla*_IMP-8_ and *bla*_VIM-1_) producers. In 2010, no other carbapenemase genes such as *bla*_KPC_, *bla*_NDM-1_, or *bla*_OXA-48-type_ were detected. However, in 2012, 22.3% (55/247) of CnSKP were carbapenemase producers with *bla*_KPC-2_ being detected in 41 isolates (16.6%). NDM-1 CnSKP also appeared in 2012. The clonal spread of ST11 KPC-2-producing *K. pneumoniae* is occurring with alarming speed, and public-health authorities should be alerted to the potential dissemination of NDM-1 as well.

KPC-KP was not reported in Taiwan until 2011. Chung *et al.* reported the first case of KPC-2-KP ventilator-associated pneumonia with bacteraemia, in a businessman working in Zhejiang Province, China, when he was transferred to Taiwan for medical management [14]. Lee *et al.* described an outbreak of 16 KPC-2-KP in 4 hospitals in northern Taiwan between June and September 2011 [26]. Although our study did not include the strains of CnSKP in 2011, the report of Lee *et al* [26] could be speculated as the origin of ST11 KPC-2-KP in Taiwan. Our study also showed that rapid intra-hospital and inter-hospital dissemination of ST11 KPC-2-KP continued in Taiwan in 2012. The difference between our study and Lee’s study was that we had enrolled different levels of hospitals in northern, central, southern, and eastern Taiwan and we had showed recent molecular basis for the increasing carbapenem resistance among *K. pneumoniae.* Through this surveillance, we found that ST11 KPC-2-KP had appeared not only in northern Taiwan but also in central and southern Taiwan. During the course of our study, ST11 KPC-2-KP became an endemic problem in Taiwan.

In 2010, the CLSI published new MIC and disk diffusion breakpoints for Enterobacteriaceae [27]. The new MIC breakpoints are 1 to 3 doubling dilutions lower than the original breakpoints (the breakpoint for carbapenems was 4 times lower for imipenem, meropenem, and doripenem and 8 times lower for ertapenem). These changes in the susceptibility breakpoints represent more sensitive criteria for the detection of carbapenem-resistant Enterobacteriaceae and eliminate the need for the detection of carbapenemase activity for making treatment decisions. By using the interpretive criteria with lower MIC breakpoints for carbapenem, the advantage is that laboratories will not miss a carbapenemase producer, while the disadvantage is that organisms having carbapenem resistance under these criteria are probably those with outer membrane porin loss combined with AmpC β-lactamase or ESBL, but not true carbapenemase producers in areas where the burdens of carbapenemase are low. Whether these changes will improve the care outcome of Enterobacteriaceae-infected patients or reduce the potential of carbapenemase needs further evaluation.

According to our results, ST11 was the epidemic clone of the KPC-2-KP in Taiwan in 2012. *K. pneumoniae* ST11 was first reported in France, and it has been reported around the world, including in the USA, Brazil [28], most countries in Europe [29], and Asia [30]. ST11 is known to be associated with different ESBLs, primarily CTX-M-15, CTX-M-14, and SHV-5 [30], [31]. ST11 is the most predominant type of ESBL-producing *K. pneumoniae* in Asian countries [32].

There is a single-locus difference between ST11 and ST258; they both belong to the clonal complex CC92 and are considered to be epidemic clones of *K. pneumoniae* with multi-drug resistance worldwide [33]. ST258 has been reported as the predominant clone of KPC-KP in the USA [34], Israel [35], and Italy [36]. KPC-producing *K. pneumoniae* ST11 have been reported as the predominant clones of carbapenem-resistant *K. pneumoniae* in China [37], Singapore [38], and the UK [39]. ST11 was also reported as a common ST for NDM-1-producing *K. pneumoniae* isolates [40]. ST11 was also reported to be associated with IMP-type metallo-β-lactamase-producing *K. pneumoniae* in Taiwan between 2002 and 2009 [41]. ST11 may be the major ST among *K. pneumoniae* isolates in Taiwan. Through the acquisition of different resistance mechanisms, a stepwise increase in resistance to carbapenem and multi-drug regimens causes a high prevalence of ST11 among CnSKP.

Our study on the resistance mechanisms of CnSKP revealed that concomitance of ESBL producers was a general phenomenon. The best choice for treatment should be tigecycline or colistin. Gentamicin, amikacin, cefepime, or SXT is not adequate for treating infections with CnSKP. A resistance rate of 98% for fluoroquinolones has also been reported, and approximately half of KPC-KP isolates were resistant to gentamicin and amikacin, as reported by a study in New York [42].

There were significant differences in the susceptibility rates to meropenem, doripenem, amikacin, gentamicin and SXT between the CnSKP isolates from 2010 and 2012. The true reason for these differences is not currently clear. The possible reasons might be that the newly participating hospitals in 2012 included eight regional hospitals but only one medical center. The antibiotics consumption of meropenem and doripenem might be lower in regional hospitals and might have contributed to the lesser resistance against meropenem and doripenem in 2012 due to lesser antibiotics selective pressure. The antibiotics consumption data needs to be collected in the further study. Another possible reason might be that KPCs are found on plasmids that often carry other β-lactamases and resistance genes [8]. The KPC-2-KP isolates in 2012 should contain plasmids that had different antibiotics resistance genes compared with CnSKP in 2010. The plasmid structure of KPC-2-KP needs further evaluation to answer this question.

Even the current distribution of KPC-2-KP appears to be homogeneous in Taiwan, it is still not clear if the spread of KPC-2 is due to similar or different plasmids. Plasmid related tests (S1-PFGE, restriction fragment length polymorphism ) is helpful to evaluate it. These organisms are commonly isolated from patients in specific regions and hospitals of Taiwan, but they are not regularly found in patients from other regions. Patients colonized or infected with CnSKP may seek medical care in more than 1 hospital, and thereby serve as reservoirs that lead to the spread of CnSKP from 1 facility to another. Based on the survey results, prevention strategies should be tailored to meet the regional needs according to the frequency of CnSKP detection [43].

In conclusion, the major resistance mechanisms of CnSKP in 2010 were due to the production of AmpC-type β-lactamase or ESBLs along with the loss of outer membrane porins, and there were no KPC-producing isolates detected in 2010. In 2012, the emergence of KPC-2 was observed and its incidence increased. More efforts are necessary to prevent the prevalence of CnSKP from increasing. Antibiotic stewardship of carbapenem and infection-control measures should be enforced. National data may help clinicians to choose appropriate antibiotics and to tailor strategies for preventing clonal outbreaks.

## Acknowledgments

The authors would like to thank Dr. Sai-Cheong Lee of KCGMH, Dr. Jen-Hsien Wang of CMUH, Dr. Cong-Yu Huang of CCGMH, Dr. Zhen-Xiang Li of KCGMH, Dr. Ke Chang of KMHH, Dr. Shih-Ta Shang of Taoyuan AFGH, Dr. Zheng-Bang Guo of Taichung AFGH, Dr. Ren-Zhi Ban of KAFGH and Dr. Xin-Bai Chen of NYMUH for their participation in the Study Group of Carbapenem Resistance in *Klebsiella pneumoniae* in Taiwan.

## Supporting Information

Figure S1 **Dendrogram of carbapenem non-susceptible** ***K. pneumoniae*** **isolates in 2010.** Dendrogram of *Xba*I -digested genomic DNA of 100 carbapenem non-susceptible *K. pneumoniae* isolates collected in 2010. There is no dominated clone causing inter-hospital spread. ST type appears on the right of the figure.
(TIF)

Figure S2 **Dendrogram of carbapenem non-susceptible** ***K. pneumoniae*** **isolates in 2012.** Dendrogram of *Xba*I -digested genomic DNA of 247 carbapenem non-susceptible *K. pneumoniae* isolates collected in 2012. KPC-2-producing-producing strains are marked with grey colour.
(TIF)

Table S1 Oligonucleotide primer sequences used for amplification of genes encoding carbapenemase, AmpC β-lactamase, and ESBLs.
(DOC)

Table S2 Resistance mechanisms among carbapenem non-susceptible K. pneumoniae isolates collected in 2010 and 2012.
(DOC)
