# Supplementary figures and images for: National Surveillance Study on Carbapenem Non-Susceptible Klebsiella pneumoniae in Taiwan: The Emergence and Rapid Dissemination of KPC-2 Carbapenemase
Source: PLoS One. 2013 Jul 24;8(7):e69428. doi: 10.1371/journal.pone.0069428 (PMC3722148; doi:10.1371/journal.pone.0069428)

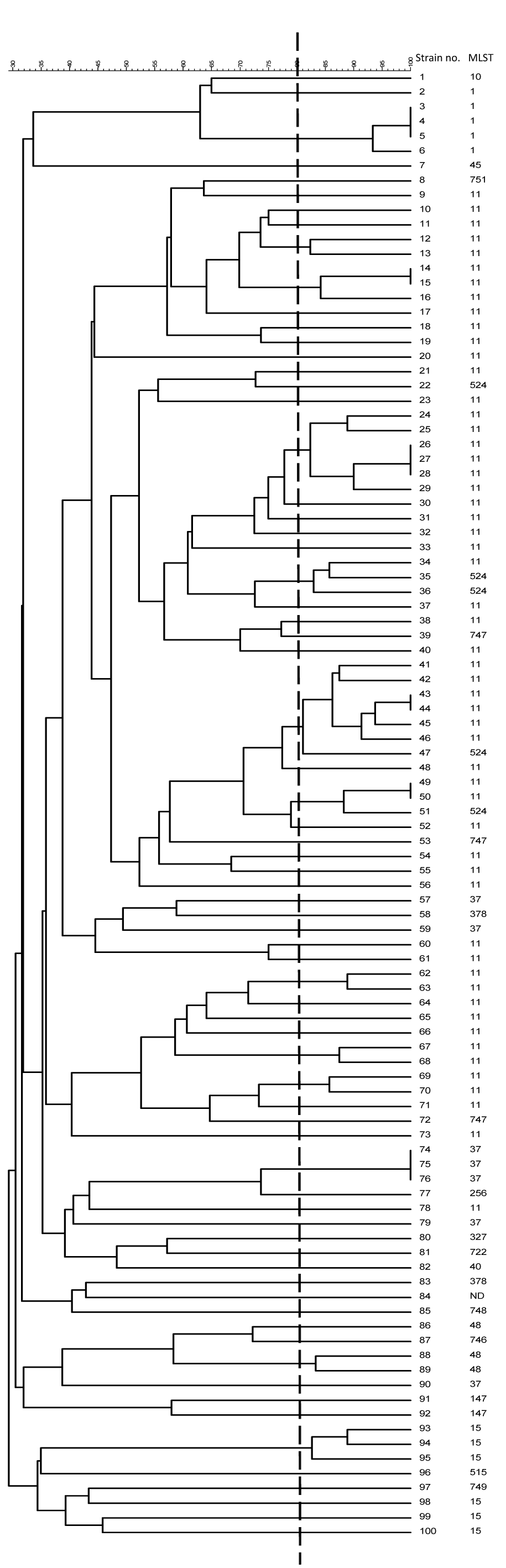

Supplement: Figure S1 — Dendrogram of carbapenem non-susceptible K. pneumoniae isolates in 2010. Dendrogram of XbaI -digested genomic DNA of 100 carbapenem non-susceptible K. pneumoniae isolates collected in 2010. There is no dominated clone causing inter-hospital spread. ST type appears on the right of the figure. (TIF) [file pone.0069428.s001.tif]

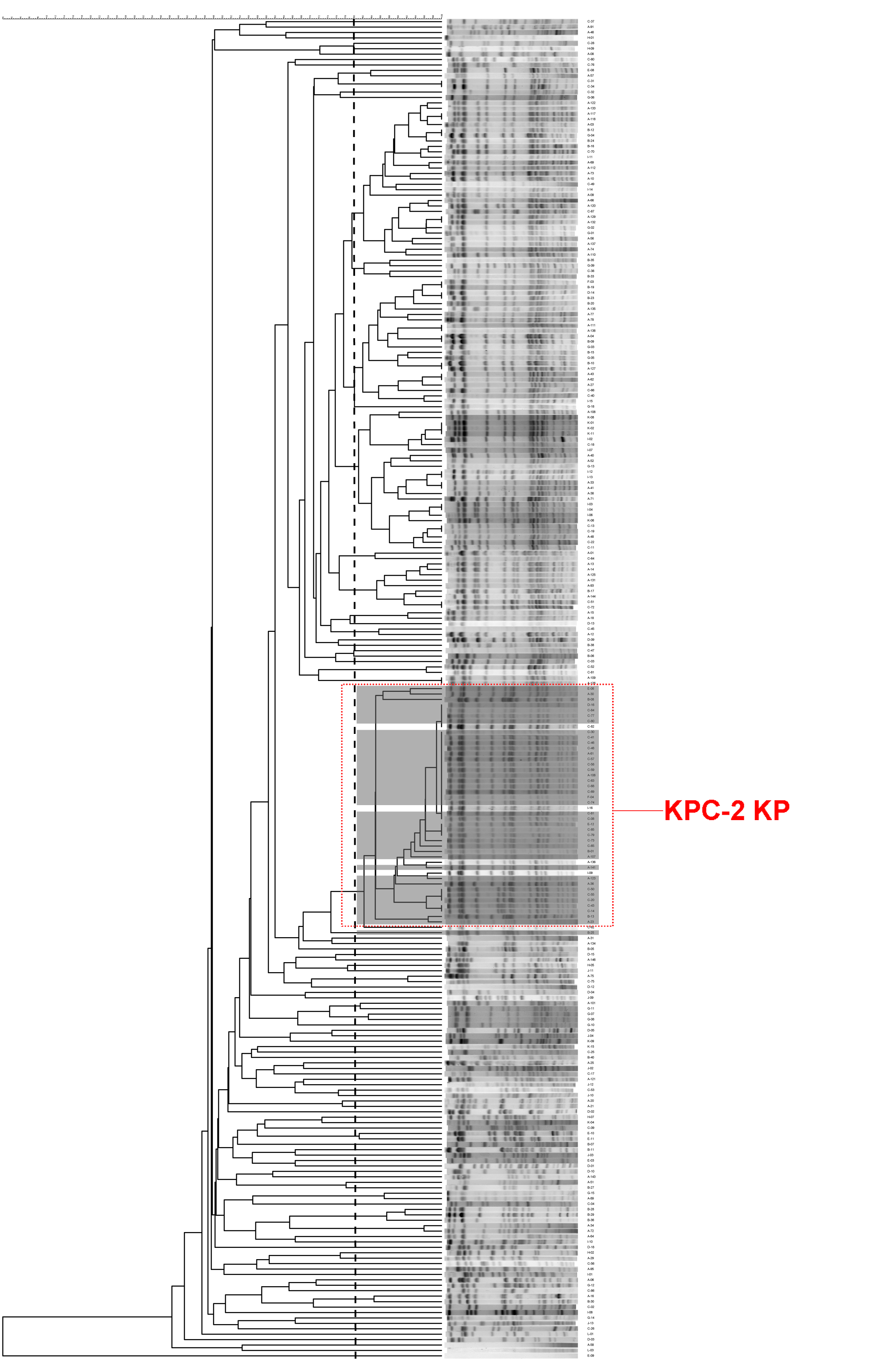

Supplement: Figure S2 — Dendrogram of carbapenem non-susceptible K. pneumoniae isolates in 2012. Dendrogram of XbaI -digested genomic DNA of 247 carbapenem non-susceptible K. pneumoniae isolates collected in 2012. KPC-2-producing-producing strains are marked with grey colour. (TIF) [file pone.0069428.s002.tif]
