# Supplementary material for: National Surveillance Study on Carbapenem Non-Susceptible Klebsiella pneumoniae in Taiwan: The Emergence and Rapid Dissemination of KPC-2 Carbapenemase
Source: PLoS One. 2013 Jul 24;8(7):e69428. doi: 10.1371/journal.pone.0069428 (PMC3722148; doi:10.1371/journal.pone.0069428)
Supplement: Table S1 — Oligonucleotide primer sequences used for amplification of genes encoding carbapenemase, AmpC β-lactamase, and ESBLs. (DOC) [file pone.0069428.s003.doc]

**Table S1: Oligonucleotide primer sequences used for amplification of genes encoding carbapenemase, AmpC β-lactamase, and ESBLs**

| PCR | Primer name | Sequence (5′-3′) | Reference |
| --- | --- | --- | --- |
| **Genes encoding class A carbapenemases** | | | |
| KPC | KPC-F | ATGTCACTGTATCGCCGTCT | 18 |
|  | KPC-R | TTTTCAGAGCCTTACTGCCC |  |
| NMC | NMC-F | GCATTGATATACCTTTAGCAGAGA | 18 |
|  | NMC-R | CGGTGATAAAATCACACTGAGCATA |  |
| IMI | IMI-F | ATAGCCATCCTTGTTTAGCTC | 18 |
|  | IMI-R | TCTGCGATTACTTATCCTC |  |
| SME | SME-F | AGATAGTAAATTTTATAG | 18 |
|  | SME-R | CTCTAACGCTAATAG |  |
| GES | GES-F | GTTTTGCAATGTGCTCAACG | 18 |
|  | GES-R | TGCCATAGCAATAGGCGTAG |  |
| **Genes encoding class B metalloenzymes** | | | |
| IMP-1 | IMP-1-F | TGAGCAAGTTATCTGTATTC | 18 |
|  | IMP-1-R | TTAGTTGCTTGGTTTTGATG |  |
| IMP-2 | IMP-2-F | GGCAGTCGCCCTAAAACAAA | 18 |
|  | IMP-2-R | TAGTTACTTGGCTGTGATGG |  |
| VIM-1 | VIM-1-F | TTATGGAGCAGCAACCGATGT | 18 |
|  | VIM-1-R | CAAAAGTCCCGCTCCAACGA |  |
| VIM-2 | VIM-2-F | AAAGTTATGCCGCACTCACC | 18 |
|  | VIM-2-R | TGCAACTTCATGTTATGCCG |  |
| SPM-1 | SPM-1F | CCTACAATCTAACGGCGACC | 18 |
|  | SPM-1R | TCGCCGTGTCCAGGTATAAC |  |
| GIM-1 | GIM-1F | AGAACCTTGACCGAACGCAG | 18 |
|  | GIM-1R | ACTCATGACTCCTCACGAGG |  |
| SIM-1 | SIM1-F | TACAAGGGATTCGGCATCG | 18 |
|  | SIM1-R | TAATGGCCTGTTCCCATGTG |  |
| NDM | NDM-F | TCTCGACAATGCCGGGTTT | In this study |
|  | NDM-R | GAGATTGCCGAGCGACTT |  |
| **Genes encoding AmpC β-lactamases** | | |  |
| CMY | CMY-F | CAAGTTTGATTCCTTGGACTCT | 44 |
|  | CMY-R | CTCATCGTCAGTTATTGCAGCT |  |
| DHA-1 | DHA-1-F | CTGATGAAAAAATCGTTATC | 45 |
|  | DHA-1-R | ATTCCAGTGCACTCAAAATA |  |
| **Genes encoding class D oxacillinases** | | |  |
| OXA-48-type | OXA-48-F | TTGGTGGCATCGATTATCGG | 46 |
|  | OXA-48-R | GAGCACTTCTTTTGTGATGGC |  |
| **Genes encoding ESBLs** | | |  |
| SHV | SHV-F | AACGGAACTGAATGAGGCGCT | 47 |
|  | SHV-R | TCCACCATCCACTGCAGCAGCT |  |
| CTX-M-1 group | CTX-M-1F | GGTTAAAAAATCACTGCGTC | 20 |
|  | CTX-M-1R | TTGGTGAGATTTTAGCCGC |  |
| CTX-M-2 group | CTX-M-2F | TGGGTTACGATTTTCGCCGC | 20 |
|  | CTX-M-2R | TGGGTTACGATTTTCGCCGC |  |
| CTX-M-9 group | CTX-M-9F | ATGGTGACAAAGAGAGTGCA | 20 |
|  | CTX-M-9R | CCCTTCGGCGATGATTCTC |  |
| TEM | TEM-F | ATGAGTATTCAACATTTCCG | 20 |
|  | TEM-R | CCAATGCTTAATCAGTGAGG |  |
