# Supplementary material for: National Surveillance Study on Carbapenem Non-Susceptible Klebsiella pneumoniae in Taiwan: The Emergence and Rapid Dissemination of KPC-2 Carbapenemase
Source: PLoS One. 2013 Jul 24;8(7):e69428. doi: 10.1371/journal.pone.0069428 (PMC3722148; doi:10.1371/journal.pone.0069428)
Supplement: Table S2 — Resistance mechanisms among carbapenem non-susceptible K. pneumoniae isolates collected in 2010 and 2012. (DOC) [file pone.0069428.s004.doc]

**Table S2. Resistance mechanisms among carbapenem non-susceptible *K. pneumoniae* isolates collected in 2010 and 2012*^a^***

| **β-lactamases^a^** |  | **Outer membrane profile** | | | | | | | | |
| --- | --- | --- | --- | --- | --- | --- | --- | --- | --- | --- |
|  |  | **2010 (N = 100)** | | | |  | **2012 (N = 247)** | | | |
|  |  | **35/36** | **△35** | **△36** | **△35/36** |  | **35/36** | **△35** | **△36** | **△35/36** |
| **Carbapenemases** |  |  |  |  |  |  |  |  |  |  |
| **KPC-2** |  | 0 | 0 | 0 | 0 |  | 1 | 35 | 2 | 3 |
| **NDM-1** |  | 0 | 0 | 0 | 0 |  | 0 | 1 | 0 | 0 |
| **IMP-8** |  | 1 | 1 | 2 | 0 |  | 0 | 6 | 0 | 0 |
| **VIM-1** |  | 0 | 1 | 0 | 1 |  | 3 | 4 | 0 | 0 |
| **AmpC** |  |  |  |  |  |  |  |  |  |  |
| **DHA-1** |  | 1 | 11 | 6 | 57 |  | 7 | 72 | 4 | 67 |
| **CMY-2** |  | 0 | 0 | 0 | 2 |  | 0 | 1 | 0 | 9 |
| **ESBLs** |  |  |  |  |  |  |  |  |  |  |
| **CTX-M-9 group** |  | 0 | 0 | 0 | 1 |  | 0 | 10 | 0 | 7 |
| **CTX-M-1 group** |  | 0 | 1 | 0 | 0 |  | 1 | 3 | 0 | 1 |
| **SHV-type (SHV-2,2A,5,12,28,31,120)** |  | 0 | 1 | 0 | 5 |  | 1 | 5 | 0 | 3 |
| **Others** |  | 0 | 3 | 1 | 5 |  | 0 | 1 | 0 | 0 |

*^a^*Most isolates have multiple β-lactamase genes. The priority for listing of β-lactamase in this table is carbapenemases, AmpC enzymes, and then ESBLs. Every isolate is listed in the table once. 35/36 indicates no deficiency of OmpK35 and OmpK36; △35 indicates deficiency of OmpK35; △36 indicate deficiency of Ompk36; and △35/36 indicates deficiency of both OmpK35 and OmpK36.
